# Prepuberal insulin secretory indices are long-term predictors of short adult stature in cystic fibrosis

**DOI:** 10.1530/EC-22-0056

**Published:** 2022-03-31

**Authors:** Alberto Battezzati, Andrea Foppiani, Gianfranco Alicandro, Arianna Bisogno, Arianna Biffi, Giorgio Bedogni, Simona Bertoli, Giulia De Carlo, Erica Nazzari, Carla Colombo

**Affiliations:** 1 International Center for the Assessment of Nutritional Status, DeFENS, University of Milan, Milan, Italy; 2 Cystic Fibrosis Center, Fondazione Istituto di Ricovero e Cura a Carattere Scientifico Ca’ Granda, Ospedale Maggiore Policlinico, University of Milan, Milan, Italy; 3 Department of Medical and Surgical Sciences, Alma Mater Studiorum University of Bologna, Bologna, Italy; 4 Internal Medicine, S. Maria delle Croci Hospital, AUSL Romagna, Ravenna, Italy; 5 Istituto Auxologico Italiano, IRCCS, Obesity Unit - Laboratory of Nutrition and Obesity Research, Department of Endocrine and Metabolic Diseases, Milan, Italy

**Keywords:** cystic fibrosis, oral glucose tolerance test, insulin secretion, beta cell glucose sensitivity

## Abstract

**Objective:**

Diabetes is a frequent comorbidity in cystic fibrosis (CF), related to multiple unfavorable outcomes. During the progression of β-cell dysfunction to diabetes, insulin deficiency could possibly reduce the anabolic support to grow even in the absence of significant glycemic derangements. To test this hypothesis, we evaluated whether prepuberal insulin secretory indices are independent predictors of adult height.

**Design:**

Observational cohort study.

**Research design and methods:**

A longitudinal analysis of 66 CF patients (33 females) from an ongoing cohort received at prepuberal age (median age of 12 years) modified 3-h oral glucose tolerance tests with 30-min insulin and C-peptide sampling, modeling of insulin secretory and sensitivity parameters, anthropometric evaluation. The latter was repeated when adults after a median follow-up of 9 years.

**Results:**

In alternative models, we found a positive association with either basal insulin secretion (mean 0.22, 95% CI 0.01, 0.44 z-scores) or prepuberal β-cell glucose sensitivity (mean 0.23, 95% CI 0.00, 0.46 z-scores) and adult height, while total insulin secretion was negatively related to adult height (mean −0.36, 95% CI −0.57, −0.15 z-scores or mean −0.42, 95% CI −0.69, −0.16 z-scores, respectively). The high total insulin secretion of low adult height patients was mainly due to late (>60 min) secretion and was associated with a worse glucose response during OGTT.

**Conclusions:**

Abnormal insulin secretion associated with high glucose response during OGTT predicts a decrease in adult height z-score. Our results suggest that insulin secretory defects in CF affect growth prior to the development of fasting hyperglycemia.

## Introduction

Insulin secretory defects in cystic fibrosis (CF) were initially shown in small-sized studies that employed various sophisticated techniques such as the hyperglycemic clamp (1, 2, 3) and intravenous glucose tolerance test (IVGTT) (4, 5, 6, 7, 8). In larger studies based on the analysis of insulin and C-peptide responses during oral glucose tolerance tests (OGTTs). These secretory defects were found to be frequent, also in normotolerant CF patients who compensate with an increased insulin sensitivity (9, 10, 11, 12, 13) and to worsen with advancing age, as we found in the population followed at the CF Center in Milan (14), in which nomograms for OGTT-derived insulin secretory parameters were produced to explore their clinical associations and significance in prospective studies.

The progressive deterioration in insulin secretion eventually leads to CF-related diabetes (CFRD), whose prevalence climbs toward 50% in adult patients (15). CFRD, in turn, has been associated with other multiple unfavorable outcomes, ranging from deterioration of nutritional status, pulmonary function and increased mortality (15). Specifically, CFRD prevents patients from achieving their full height genetic potential (16), which is a severe consequence per se, being related to mortality (17). However, linear growth reduction precedes CFRD (16, 18) and cannot be reversed with intensive insulin treatment once diabetes is diagnosed (19), suggesting that insulin secretory defects may play a negative role.

The concept that subclinical insulin secretory defects may affect linear growth independently from hyperglycemia has been suggested by a small-sized study in CF (20), but further evidence arises from studies in type 1 diabetes and in healthy subjects. According to mechanistic studies, insulin is one of the main regulators of growth hormone (GH)/insulin-like growth factor 1 (IGF-1) axis (21), frequently involved in growth failure associated with several chronic conditions including CF. In the past, subjects with uncontrolled type 1 diabetes may have suffered dwarfism associated with Mauriac Syndrome (22). Currently, the degree of glycemic control may still impact linear growth (23, 24), but an independent protective effect of residual β cell function has been documented (25). Furthermore, an earlier work in healthy adult males (26) showed that insulin responses to glucose are associated with height.

Evidence mentioned above supports the idea that the slowly progressing β cell dysfunction in CF possibly reduces the anabolic support to grow even in the absence of significant glycemic derangements. To test this hypothesis, we evaluated whether prepuberal insulin secretory indices measured during OGTT are independent predictors of adult height, measured approximately 10 years later.

## Research design and methods

### Design

This was a prospective cohort study with a mean follow-up of 9 years.

### Setting

Between 2003 and 2013, the CF Center of Milan offered participation in this study to all CF patients at prepuberal age and regularly followed up with clinical and laboratory assessment, including an annual OGTT. The study was approved by the Milano Area 2 ethical committee (n. 452, 556 and 777). Patients were informed about the purpose of the study and gave permission to include their clinical and laboratory data in this research.

### Patient inclusion criteria

OGTTs were performed between the ages of 8.5 and 12.9 years in CF patients of both sexes who were prepuberal (according to menarche age and or Tanner Stage), clinically stable in the previous 3 weeks (absence of major clinical events including pulmonary exacerbations, no change in their habitual treatment regimen including the introduction of antibiotics or steroids) and had not previously received a CFRD diagnosis or treatment with insulin or oral hypoglycemic agents in the previous 6 months. Patients who had received or were listed for a lung or liver transplant began taking GH replacement, modulator therapy, or corticosteroids treatment at any time prior to the end of follow-up were also excluded. OGTTs were performed between 2003 and 2013. All patients who reached 18 years of age and had their height recorded at the time of the study were included; the last available height was used and the end of follow-up was set at that date. Sixty-six patients complied with these criteria and were included in this analysis.

### Assessment at baseline

All subjects received a 3-h OGTT (1.75 g/kg, maximum 75 g) sampling at baseline and at 30-min intervals the plasma glucose, serum insulin and C-peptide concentrations. Pulmonary function (FEV1% and FVC) and nutritional status (height, weight and BMI) were also measured, as well as parents’ height from which mid-parental height was calculated.

### Analytical methods

Plasma glucose was measured on fluoride plasma samples (Gluco-quant; Roche/Hitachi analyzer; Roche Diagnostics), and the other analytes were measured by commercial assays (ECLIACobas C6000; Roche Diagnostics).

### OGTT analysis

Based on the plasma glucose concentrations, patients were assigned to one of the following categories of glucose tolerance (15): normal glucose tolerance, normal glucose tolerance with impaired fasting glucose, indeterminate glucose tolerance, impaired glucose tolerance, cystic fibrosis-related diabetes without fasting hyperglycemia and cystic fibrosis-related diabetes with fasting hyperglycemia. Insulin secretory and insulin sensitivity parameters were derived from the modeling of glucose, insulin and C-peptide profiles as we previously described (14, 27, 28).

Specifically, insulin secretion was calculated from glucose and C-peptide concentrations by regularized least squares (20). Regularization involves the choice of smoothing factors that were selected to obtain glucose and C-peptide model residuals with SDs close to the expected measurement error (~1% for glucose and ~4% for C-peptide). Insulin secretion rates were calculated from the model every 5 min. The integral of insulin secretion during the 3-h OGTT was denoted as total insulin output. β-cell glucose sensitivity represents the responsiveness of insulin secretion to glucose.

Insulin sensitivity was determined in the basal state using the homeostasis model assessment from basal levels (HOMA) index and during the OGTT with the 3-h oral glucose tolerance test-based index of insulin sensitivity (OGIS) index (29).

Insulin clearance was calculated in the fasting state as the ratio between fasting insulin secretion and fasting insulin concentration and during the OGTT as the ratio between the integral of insulin secretion and that of insulin concentration.

The above parameters were also evaluated according to reference values for sex and age recently developed in the CF Center of Milan (14).

### Statistical analysis

Continuous variables are reported as median (25th, 75th percentile), and categorical variables are reported as count (percentages). Comparisons across groups were carried out using the Wilcoxon rank-sum test with continuity correction (for continuous variables) or Pearson’s chi-squared test or Fisher’s exact test (for categorical variables).

Ordinary least square linear models were used to describe the relationship between indices of insulin and glucose response to OGTT and adult height z-score. Models were adjusted for sex (categorical; 0 = female, 1 = male), pancreatic insufficiency (defined by fecal elastase (30)) (dichotomous; 0 = pancreatic sufficiency, 1 = pancreatic insufficiency), target height z-score. Different models were fitted, each including a single index of insulin and glucose response to OGTT as predictors: β-cell glucose sensitivity, integral of total insulin secretion (adjusted for basal insulin secretion), OGTT insulin clearance (adjusted for basal insulin clearance), HOMA index and OGIS index, and OGTT glucose AUC (adjusted for basal glucose). A final complete model including all the indices was also fitted. The association between OGTT-derived indices and adult height was expressed as average predicted changes in height z-score for one interquartile increase of the index.

Statistical analyses were performed in R 4.1.0 (31).

## Results

Baseline (prepuberal) patient characteristics are reported in Table 1. Of the subjects included, 57 (86%) were pancreatic insufficient (29 females and 28 males) and all were consuming pancreatic enzymes replacement therapy. None of the patients were assuming CFTR modulator therapy. Z-scores of weight, height and BMI were not significantly different between sexes, but they were reduced when compared to the general population. Most patients (68%) had normal glucose tolerance but β cell glucose sensitivity and insulin secretion were lower in males (although differences in β cell glucose sensitivity did not reach statistical significance).

**Table 1:** Baseline patient characteristics by sex.

Characteristic	Overall, *n* = 66	Female, *n* = 33	Male, *n* = 33	*P*-value
Age (years)	11.95 (10.92, 12.49)	11.51 (10.67, 12.45)	12.25 (11.25, 12.59)	0.054
CFTR mutation				0.7
F508del homozygous	25 (38%)	11 (33%)	14 (42%)	
F508del heterozygous	27 (41%)	15 (45%)	12 (36%)	
Other	14 (21%)	7 (21%)	7 (21%)	
Meconium ileus	11 (17%)	5 (15%)	6 (18%)	0.7
Pancreatic insufficient	57 (86%)	29 (88%)	28 (85%)	>0.9
Liver disease	24 (36%)	10 (30%)	14 (42%)	0.3
Pseudomonas aeruginosa				0.8
No	19 (29%)	11 (33%)	8 (24%)	
Intermittent	43 (65%)	20 (61%)	23 (70%)	
Chronic	4 (6.1%)	2 (6.1%)	2 (6.1%)	
Burkholderia cepacia	1 (1.5%)	0 (0%)	1 (3.0%)	>0.9
Height z-score	−0.39 (−0.62, −0.17)	−0.47 (−0.69, −0.16)	−0.33 (−0.53, −0.19)	0.3
Weight z-score	−0.48 (−0.69, −0.19)	−0.51 (−0.73, 0.28)	−0.37 (−0.61, −0.13)	0.3
Body mass index z-score	−0.42 (−0.62, −0.12)	−0.46 (−0.68, −0.13)	−0.37 (−0.58, −0.11)	0.5
Body mass index category				>0.9
Underweight	0 (0%)	0 (0%)	0 (0%)	
Normal weight	66 (100%)	33 (100%)	33 (100%)	
Overweight	0 (0%)	0 (0%)	0 (0%)	
Obesity	0 (0%)	0 (0%)	0 (0%)	
FEV1 (% of predicted)	87 (81, 95)	87 (82, 95)	87 (81, 97)	>0.9
FVC (% of predicted)	95 (87, 101)	95 (86, 100)	95 (87, 101)	0.7
HbA1C (%)	5.90 (5.50, 6.20)	5.80 (5.60, 6.60)	5.90 (5.40, 6.10)	0.4
Glucose tolerance				**0.039**
Normal glucose tolerance	45 (68%)	18 (55%)	27 (82%)	
Normal glucose tolerance with impaired fasting glucose	0 (0%)	0 (0%)	0 (0%)	
Indeterminate glucose tolerance	10 (15%)	8 (24%)	2 (6.1%)	
Impaired glucose tolerance	8 (12%)	4 (12%)	4 (12%)	
Cystic fibrosis related diabetes without fasting hyperglycemia	3 (4.5%)	3 (9.1%)	0 (0%)	
Cystic fibrosis related diabetes with fasting hyperglycemia	0 (0%)	0 (0%)	0 (0%)	
Homeostatic model assessment for insulin resistance	1.09 (0.62, 1.56)	1.10 (0.51, 1.46)	1.09 (0.64, 1.58)	0.7
Oral glucose insulin sensitivity index (mL min−1 m−2)	539 (497, 621)	560 (491, 621)	529 (507, 621)	0.9
β cell glucose sensitivity (pmol×min−1×m−2×mM−1)	79 (50, 106)	89 (52, 124)	71 (45, 94)	0.10
Basal insulin secretion (pmol min−1 m−2)	82 (59, 96)	90 (67, 105)	72 (49, 90)	**0.007**
Integral of OGTT insulin secretion (nmol m−2)	55 (43, 68)	57 (50, 70)	46 (37, 64)	**0.013**
Basal insulin clearance (L min−1 m−2)	2.22 (1.79, 2.95)	2.43 (2.03, 3.64)	2.19 (1.64, 2.46)	**0.014**
OGTT insulin clearance (L min−1 m−2)	1.66 (1.30, 1.97)	1.75 (1.44, 2.51)	1.61 (1.27, 1.85)	0.10
Basal glucose (mg dL)	80 (72, 89)	80 (76, 89)	81 (71, 89)	0.7
OGTT glucose AUC (mg dL min−1)	20,452 (18,375, 24,896)	21,615 (18,825, 27,510)	20,400 (18,120, 22,755)	0.2

Bold indicates statistical significance.

Table 2 reports differences in anthropometric parameters and respiratory status evaluated at prepuberal and adult ages. Median follow-up was 9 years, during which 9⁄66 (14%) patients became diabetic and were on insulin at the end of follow-up (all of them were among pancreatic insufficient patients, 9⁄57 (16%)). Adult height z-score was lower than target height z-score by 0.22 z-scores (CI: 0.04, 0.40; *P*  = 0.017) and 11 (17%) patients were underweight at the end of follow-up. Adult FEV1 (% of predicted) was −13% (CI: −20, −6.8; *P*  < 0.001) compared to prepuberal age.

**Table 2:** Differences for age, nutritional and pulmonary status between follow-up (adult age) and baseline (prepuberal age). Median follow-up of 9 years.

Characteristic	Baseline, *n* = 66	Follow-up, *n* = 66	Difference	95% CI	*P*-value
Height z-score	−0.39 (−0.62, −0.17)	−0.30 (−0.78, 0.10)	0.09	0.01, 0.20	0.078
Weight z-score	−0.48 (−0.69, −0.19)	−0.31 (−0.59, 0.08)	0.20	0.09, 0.30	<0.001
Body mass index z-score	−0.42 (−0.62, −0.12)	−0.13 (−0.42, 0.14)	0.25	0.15, 0.35	<0.001
FEV1 (% of predicted)	87 (79, 101)	79 (55, 96)	−13	−20, −6.8	<0.001

Bold indicates statistical significance.

Table 3 shows results from the predictive models of adult height z-score. No significant differences were found between sexes and patients with and without pancreatic insufficiency. The model testing the association between glucose response and adult height showed a negative association for OGTT glucose AUC adjusted for basal glucose, although the association did not reach statistical significance. The insulin secretion model showed a positive association of adult height z-score with basal insulin secretion and a negative association with total insulin secretion, both statistically significant. In the model including all OGTT parameters, β-cell glucose sensitivity was positively associated with adult height, while total insulin secretion maintained the negative association.

**Table 3:** Association between prepuberal OGTT-based indices and adult height.

Characteristic	Glucose model			Insulin model			β cell sensitivity model			Insulin resistance model			Insulin clearance model			Complete model		
	Beta	95% CI	*P*-value	Beta	95% CI	*P*-value	Beta	95% CI	*P*-value	Beta	95% CI	*P*-value	Beta	95% CI	*P*-value	Beta	95% CI	*P*-value
(Intercept)	0.05	−0.81, 0.91	>0.9	0.23	−0.33, 0.79	0.4	−0.43	−1.0, 0.11	0.12	−0.59	−1.6, 0.39	0.2	−0.05	−0.46, 0.36	0.8	−0.20	−2.2, 1.8	0.8
Sex																		
Female	–	–	–	–	–	–	–	–	–	–	–	–	–	–	–	–	–	–
Male	−0.07	−0.33, 0.18	0.6	−0.11	−0.36, 0.14	0.4	0.01	−0.24, 0.27	>0.9	−0.03	−0.29, 0.22	0.8	−0.07	−0.34, 0.21	0.6	−0.16	−0.45, 0.12	0.3
Pancreatic insufficiency	0.01	−0.38, 0.39	>0.9	−0.09	−0.44, 0.26	0.6	0.01	−0.39, 0.40	>0.9	−0.08	−0.46, 0.30	0.7	−0.07	−0.45, 0.32	0.7	0.05	−0.34, 0.43	0.8
Target height z-score	0.63	0.34, 0.93	<0.001	0.66	0.38, 0.93	<0.001	0.64	0.35, 0.94	<0.001	0.64	0.34, 0.94	<0.001	0.65	0.33, 1.0	<0.001	0.65	0.35, 0.95	<0.001
Basal glucose (IQR increase)	0.06	−0.11, 0.22	0.5													−0.01	−0.22, 0.21	>0.9
OGTT glucose AUC (IQR increase)	−0.16	−0.32, 0.01	0.060													0.10	−0.11, 0.31	0.4
Basal insulin secretion (IQR increase)				0.22	0.01, 0.44	**0.039**										0.06	−0.25, 0.37	0.7
Integral of OGTT insulin secretion (IQR increase)				−0.36	−0.57, −0.15	**0.001**										−0.42	−0.69, −0.16	**0.002**
β-cell glucose sensitivity (IQR increase)							0.12	−0.05, 0.28	0.2							0.23	0.00, 0.46	**0.046**
Homeostatic model assessment for insulin resistance (IQR increase)										0.07	−0.07, 0.22	0.3				0.12	−0.09, 0.33	0.2
Oral glucose insulin sensitivity index (IQR increase)										0.08	−0.11, 0.26	0.4				0.03	−0.20, 0.26	0.8
Basal insulin clearance (IQR increase)													0.00	0.00, 0.00	0.4	0.00	0.00, 0.00	0.5
OGTT insulin clearance (IQR increase)													−0.04	−0.09, 0.01	0.13	−0.03	−0.08, 0.02	0.2

Bold indicates statistical significance.

**Figure 1:**
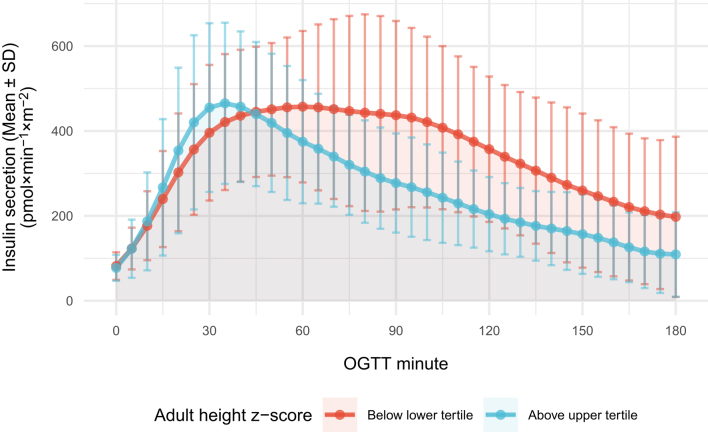
Prepuberal insulin secretion profiles of the patients in the extreme tertiles of adult height.

Figure 1 shows insulin secretion profiles for patients with an adult height below the lower tertile and those above the upper tertile. Patients with a low adult height reached peak insulin secretory rate significantly later than patients above the upper tertile of adult height (mean peak minute 70 vs 47; mean difference 22 min; 95% CI 8, 37; *P*  = 0.009), and secreted more insulin in the second and third hour of the test (total insulin secretion AUC between 60 and 180 OGTT minutes 41458 vs 26154; mean difference 15305; 95% CI 7316, 23292; *P*  ≤ 0.001).

## Discussion

The main finding of this study is that insulin secretory indices and possibly OGTT glucose response measured at prepuberal age represent long-term predictors of adult height in patients with CF.

We selected a subgroup of patients followed at the CF center of Milan, in a narrow age range and all in the prepubertal stage. The OGTTs were performed when CFTR modulators were not yet introduced in clinical practice. We have previously shown that the distribution of insulin secretory parameters measured by OGTT is shifted toward lower values, compared to healthy control subjects, also in normotolerant CF patients (14).

Patients’ height and BMI were generally reduced already at the prepuberal age (Table 2), as previously described in the Italian CF population (32). We evaluated the effect of the disease on adult height adjusting for each patient’s target height (mid-parental height), to approximately account for maximum adult height potential. We found a median interquartile range (IQR) difference of −0.22 (−0.51, 0.09) z-scores between target height and achieved adult height, with no significant difference between sexes (*P*  = 0.6). Overall, our analysis supports the idea that insulin secretory indices are related to defective growth and are predictive of a reduced adult height.

Several factors may concur to explain this relationship. In our study, adult height was positively related to β cell glucose sensitivity, but it was negatively related to total insulin secretion. In the context of CF, β-cell glucose sensitivity is an indicator of a healthy endocrine pancreas that could have favored optimal growth. On the other hand, the association with total insulin secretion was negative, although with a wide CI. While limitations in the sample size surely contributed to this uncertainty, in part it may be due to modeling the relationship between insulin secretion and adult height as linear (another limitation due to the limited sample size). It is plausible, and it was our original hypothesis, that higher insulin should promote growth through the GH/IGF-1 axis, but our data suggest that this is limited to higher insulin secreted early during the OGTT. A physiological response to an OGTT sees a timely and early elevation of insulin levels that peaks between 30 and 60 OGTT min (33). In our sample, the highest total insulin secretion levels were not obtained through an elevation of insulin secretion in the early stages of OGTT, but through a delayed response that compensated for increased glucose concentration in the late part of the OGTT.

We did not provide a mechanistic explanation for how a delayed and lately increased insulin secretion may have negatively affected growth. To date, the available studies are not conclusive but may support several speculations. The GH/IGF-1 axis is one of the main regulators of linear growth in children (34). GH promotes growth indirectly by inducing the release of insulin-like growth factor 1 (IGF-1) from the liver and the local secretion of IGF-1 from cartilage cells in the growth plates of the long bones, but it also displays a direct, IGF-1-independent, effect (35). Sustained insulin levels stimulate IGF-1 secretion (36), but may also lead to the inhibition of GH secretion (37) and signaling (38), playing an overall detrimental effect on growth. It is therefore plausible that a pulsatile rather than continuous insulin secretion may be required for optimal growth.

The negative association between adult height and total insulin secretion was consistent with what we previously noted (13), i.e. that hyperglycemia can stimulate total insulin output (and insulin and C-peptide AUCs) to levels similar or even greater than in healthy subjects. This finding has practical implications, because the absolute insulin secretion rates or the insulin or C-peptide concentrations or AUCs may not accurately describe β-cell function defects.

Indeed, we showed in Fig. 1 that the main determinant of a high total insulin secretion in subjects below the lower tertile of adult height z-score was a late (>60 min) insulin secretion. Our data suggest that an increased total insulin secretion that is delayed because of a failing beta-cell sensitivity to glucose, not only is associated with a deteriorated glucose tolerance but is also associated with growth failure.

Our data do not exclude an adverse role of hyperglycemia on linear growth. High glucose levels in the late part of OGTT are probably the driver for this late insulin secretion and in the simple model of glucose response, OGTT glucose response, but not basal glucose, was negatively associated with adult height, at a nearly significant level. It is already known that CFRD has a large effect on nutritional status (39) and that hyperglycemia, consequent to insulin secretory defects and insulin resistance, could be a causative factor as it has been associated with defective linear growth (23). In theory, hyperglycemia could be responsible for either nutrient renal losses or reduced immune defenses especially in the respiratory tract (40), even though hyperglycemia is generally not accompanied by reduced linear growth in the absence of insulin secretory defects (as in type 2 diabetes).

We could not determine if the negative association with adult height was due to the abnormal insulin response or due to hyperglycemia. Our complete model seems to favor the differences in insulin secretion as an explanatory variable for differences in adult height, while glucose response could not explain an additional amount of variance in the outcome (although this may also be due to sample size limitations).

Insulin therapy is usually effective in glucose control, but only approximate insulin secretion from the pancreas; the fact that it cannot completely normalize suboptimal growth, both in CF (21) and type 1 diabetes (41), may suggest that an optimal insulin secretion is more important that glucose control for growth. Furthermore, several findings stand against the idea that hyperglycemia of diabetes has driven the observed relationship of insulin secretory indices with adult height. In agreement with what has been already described in our group (42), future diabetics were already in shorter stature at prepubertal age prior to the development of CFRD, which suggests that overt hyperglycemia is not the cause of short stature. In addition, in this study, when accounting for insulin secretory parameters, neither insulin sensitivity nor OGTT glucose response was associated with height.

Finally, more severe genetic defects may promote a greater inflammatory phenotype which may be associated with reduced linear growth and even growth arrest (43). Greater inflammation would cause greater insulin resistance (44), further exacerbated by the use of steroid drugs. Against this hypothesis, we did not find a negative association between height with insulin sensitivity. This trait is highly variable over time in CF (45), thus it is very difficult for insulin resistance to be a long-term predictor of adult height. Furthermore, we have shown that insulin sensitivity and β cell function may be inversely related to CF (13). Also for this reason it is suggested that insulin secretory defects are related to linear growth independently from the inflammatory phenotype in CF patients.

If confirmed, this association is likely to be clinically relevant. First, height is an independent predictor of mortality in CF (17). Secondly, insulin secretory defects can be treated (intervention studies remain to be done) but they are presently not until the diagnosis of CFRD (15). Several trials have tested the effects of insulin administration in the absence of hyperglycemia, but the evidence is inconclusive (46). Moran (47) and Minicucci (48) performed controlled studies and observed effects on BMI, however for their relatively short duration and small size they were not powered to detect effects on height. Finally, this is a natural history study that may set a standard to compare the effect of emerging CFTR modulator therapies.

The main strengths of this study are the inclusion of patients in a strict age range prior to puberty, the measurement of parameters of β-cell function ranging from the simplest ones (insulin concentrations) to more sophisticated ones (β-cell glucose sensitivity, based on c-peptide and unaffected by hepatic insulin clearance), and in the prospective design that allowed a long-term follow-up. The main weakness of this study relates to the limited sample size of patients followed at a single center. The number of patients who reached 18 years of age and had their height recorded at the time of the study resulted in the sample size, and no a priori calculation of sample size was performed. While this study may provide an early signal of the association of prepuberal insulin secretory indices with short adult stature in cystic fibrosis, these findings should be confirmed in larger studies. Furthermore, the GH/IGF-1 axis was not explored.

In conclusion, we have followed for almost 10 years a cohort of CF patients who had received an OGTT and measured glucose and insulin secretory responses and insulin sensitivity. Our data indicate that final height is compromised in Cystic Fibrosis. Reduced height is not related to a reduction in the absolute amount of insulin secreted in response to glucose. Nonetheless, reduced height is related to a reduced beta-cell glucose sensitivity, a defect in the quality of insulin secretion that causes a delayed and lately sustained insulin response to glucose. This study does not provide a mechanistic explanation but suggests that altered insulin dynamics may play a role in the reduction of adult height. The possible interference of altered insulin secretory dynamics in the complex regulation of the GH/IGF-1 axis remains to be clarified.

## Declaration of interest

The authors declare that there is no conflict of interest that could be perceived as prejudicing the impartiality of the research reported.

## Funding

This work was supported by grants from the Italian Cystic Fibrosis Research Foundation FFC 16/2005, FFC 21/2013, FFC 20/2016, and FFC 24/2019.

## Author contribution statement

A Ba wrote the manuscript. A Bif researched data. A Bis researched data and reviewed/edited the manuscript. A F performed statistical analysis and reviewed/edited the manuscript. C C reviewed/edited the manuscript. E N researched data. G A reviewed statistical analysis and reviewed/edited the manuscript. G B reviewed statistical analysis and reviewed/edited the manuscript. G D C researched data. S B reviewed/edited the manuscript.

## References

[1] Rakotoambinina B Delaisi B Laborde K Silly C De Blic J Lenoir G Robert JJ. Insulin responses to intravenous glucose and the hyperglycemic clamp in cystic fibrosis patients with different degrees of glucose tolerance. Pediatric Research 1994 36 667–671. (10.1203/00006450-199411000-00024) 7877889

[2] Moran A Pyzdrowski KL Weinreb J Kahn BB Smith SA Adams KS Seaquist ER. Insulin sensitivity in cystic fibrosis. Diabetes 1994 43 1020–1026. (10.2337/diab.43.8.1020) 8039595

[3] Austin A Kalhan SC Orenstein D Nixon P Arslanian S. Roles of insulin resistance and beta-cell dysfunction in the pathogenesis of glucose intolerance in cystic fibrosis. Journal of Clinical Endocrinology and Metabolism 1994 79 80–85. (10.1210/jcem.79.1.8027259) 8027259

[4] Moran A Diem P Klein DJ Levitt MD Robertson RP. Pancreatic endocrine function in cystic fibrosis. Journal of Pediatrics 1991 118 715–723. (10.1016/s0022-3476(0580032-0) 2019925

[5] Hinds A Sheehan AG Parsons HG. Tolbutamide causes a modest increase in insulin secretion in cystic fibrosis patients with impaired glucose tolerance. Metabolism: Clinical and Experimental 1995 44 13–18. (10.1016/0026-0495(9590283-x) 7854158

[6] Holl RW Heinze E Wolf A Rank M Teller WM. Reduced pancreatic insulin release and reduced peripheral insulin sensitivity contribute to hyperglycaemia in cystic fibrosis. European Journal of Pediatrics 1995 154 356–361. (10.1007/BF02072102) 7641765

[7] Garagorri JM Rodriguez G Ros L Sanchez A. Early detection of impaired glucose tolerance in patients with cystic fibrosis and predisposition factors. Journal of Pediatric Endocrinology and Metabolism 2001 14 53–60. (10.1515/jpem.2001.14.1.53) 11220706

[8] Tofé S Moreno JC Máiz L Alonso M Escobar H Barrio R. Insulin-secretion abnormalities and clinical deterioration related to impaired glucose tolerance in cystic fibrosis. European Journal of Endocrinology 2005 152 241–247. (10.1530/eje.1.01836) 15745932

[9] Bismuth E Laborde K Taupin P Velho G Ribault V Jennane F Grasset E Sermet I De Blic J Lenoir G***et al***. Glucose tolerance and insulin secretion, morbidity, and death in patients with cystic fibrosis. Journal of Pediatrics 2008 152 540–545, 545.e1. (10.1016/j.jpeds.2007.09.025) 18346512

[10] Anzeneder L Kircher F Feghelm N Fischer R Seissler J. Kinetics of insulin secretion and glucose intolerance in adult patients with cystic fibrosis. Hormone and Metabolic Research 2011 43 355–360. (10.1055/s-0031-1275270) 21448848

[11] Elder DA Wooldridge JL Dolan LM D’Alessio DA. Glucose tolerance, insulin secretion, and insulin sensitivity in children and adolescents with cystic fibrosis and no prior history of diabetes. Journal of Pediatrics 2007 151 653–658. (10.1016/j.jpeds.2007.05.012) 18035147

[12] Yung B Noormohamed FH Kemp M Hooper J Lant AF Hodson ME. Cystic fibrosis-related diabetes: the role of peripheral insulin resistance and β-cell dysfunction. Diabetic Medicine 2002 19 221–226. (10.1046/j.1464-5491.2002.00666.x) 11963922

[13] Battezzati A Mari A Zazzeron L Alicandro G Claut L Battezzati PM Colombo C. Identification of insulin secretory defects and insulin resistance during oral glucose tolerance test in a cohort of cystic fibrosis patients. European Journal of Endocrinology 2011 165 69–76. (10.1530/EJE-10-1003) 21502328

[14] Battezzati A Bedogni G Zazzeron L Mari A Battezzati PM Alicandro G Bertoli S Colombo C. Age-and sex-dependent distribution of OGTT-related variables in a population of cystic fibrosis patients. Journal of Clinical Endocrinology and Metabolism 2015 100 2963–2971. (10.1210/jc.2015-1512) 26057180

[15] Moran A Pillay K Becker D Granados A Hameed S Acerini CL. ISPAD clinical practice consensus guidelines 2018: management of cystic fibrosis-related diabetes in children and adolescents. Pediatric Diabetes 2018 19 (Supplement 27) 64–74. (10.1111/pedi.12732) 30094886

[16] Terliesner N Vogel M Steighardt A Gausche R Henn C Hentschel J Kapellen T Klamt S Gebhardt J Kiess W***et al***. Cystic-fibrosis related-diabetes (CFRD) is preceded by and associated with growth failure and deteriorating lung function. Journal of Pediatric Endocrinology and Metabolism 2017 30 815–821. (10.1515/jpem-2017-0005) 28245190

[17] Vieni G Faraci S Collura M Lombardo M Traverso G Cristadoro S Termini L Lucanto MC Furnari ML Trimarchi G***et al***. Stunting is an independent predictor of mortality in patients with cystic fibrosis. Clinical Nutrition 2013 32 382–385. (10.1016/j.clnu.2012.08.017) 22974535

[18] Cheung MS Bridges NA Prasad SA Francis J Carr SB Suri R Balfour-Lynn IM. Growth in children with cystic fibrosis-related diabetes. Pediatric Pulmonology 2009 44 1223–1225. (10.1002/ppul.21127) 19894249

[19] Bizzarri C Montemitro E Pedicelli S Ciccone S Majo F Cappa M Lucidi V. Glucose tolerance affects pubertal growth and final height of children with cystic fibrosis. Pediatric Pulmonology 2015 50 144–149. (10.1002/ppul.23042) 24678051

[20] Ripa P Robertson I Cowley D Harris M Masters IB Cotterill AM. The relationship between insulin secretion, the insulin-like growth factor axis and growth in children with cystic fibrosis. Clinical Endocrinology 2002 56 383–389. (10.1046/j.1365-2265.2002.01484.x) 11940051

[21] Wong SC Dobie R Altowati MA Werther GA Farquharson C Ahmed SF. Growth and the growth hormone-insulin like growth factor 1 axis in children with chronic inflammation: current evidence, gaps in knowledge, and future directions. Endocrine Reviews 2016 37 62–110. (10.1210/er.2015-1026) 26720129

[22] Mauriac P Hepatomegaly, dwarfism, obesity and diabetes in children: Mauriac’s syndrome. Vida Nueva 1951 67 57–65. 14877009

[23] Bonfig W Kapellen T Dost A Fritsch M Rohrer T Wolf J Holl RW & Diabetes Patienten Verlaufsdokumentationssystem Initiative of the German Working Group for Pediatric Diabetology and the German Bundesministerium für Bildung und Forschung Competence Netfor Diabetes Mellitus. Growth in children and adolescents with type 1 diabetes. Journal of Pediatrics 2012 160 900.e2–903.e2. (10.1016/j.jpeds.2011.12.007) 22244464

[24] Giannini C Mohn A Chiarelli F. Growth abnormalities in children with type 1 diabetes, juvenile chronic arthritis, and asthma. International Journal of Endocrinology 2014 2014 265954. (10.1155/2014/265954) 24648838 PMC3932221

[25] Bizzarri C Benevento D Patera IP Bongiovanni M Boiani A Fusco C Cianfarani S Cappa M. Residual β-cell mass influences growth of prepubertal children with type 1 diabetes. Hormone Research in Paediatrics 2013 80 287–292. (10.1159/000355116) 24051686

[26] Alvarsson M Efendic S Grill VE. Insulin responses to glucose in healthy males are associated with adult height but not with birth weight. Journal of Internal Medicine 1994 236 275–279. (10.1111/j.1365-2796.1994.tb00796.x) 8077883

[27] Mari A Tura A Gastaldelli A Ferrannini E. Assessing insulin secretion by modeling in multiple-meal tests: role of potentiation. Diabetes 2002 51 (Supplement 1) S221–S226. (10.2337/diabetes.51.2007.s221) 11815483

[28] Mari A Schmitz O Gastaldelli A Oestergaard T Nyholm B Ferrannini E. Meal and oral glucose tests for assessment of β-cell function: modeling analysis in normal subjects. American Journal of Physiology: Endocrinology and Metabolism 2002 283 E1159–E1166. (10.1152/ajpendo.00093.2002) 12388151

[29] Mari A Pacini G Murphy E Ludvik B Nolan JJ. A model-based method for assessing insulin sensitivity from the oral glucose tolerance test. Diabetes Care 2001 24 539–548. (10.2337/diacare.24.3.539) 11289482

[30] Walkowiak J Herzig KH Strzykala K Przyslawski J Krawczynski M. Fecal elastase-1 is superior to fecal chymotrypsin in the assessment of pancreatic involvement in cystic fibrosis. Pediatrics 2002 110 e7–e7. (10.1542/peds.110.1.e7) 12093988

[31] R CoreTeam. R: A Language and Environment for Statistical Computing. Vienna, Austria: R Foundation for StatisticalComputing, 2021.

[32] Lucidi V Alghisi F Raia V Russo B Valmarana L Valmarana R Coruzzo A Beschi S Dester S Rinaldi D***et al***. Growth assessment of paediatric patients with CF comparing different auxologic indicators: a multicentre Italian study. Journal of Pediatric Gastroenterology and Nutrition 2009 49 335–342. (10.1097/MPG.0b013e31818f0a39) 19543116

[33] Hayashi T Boyko EJ Sato KK McNeely MJ Leonetti DL Kahn SE Fujimoto WY. Patterns of insulin concentration during the OGTT predict the risk of type 2 diabetes in Japanese Americans. Diabetes Care 2013 36 1229–1235. (10.2337/dc12-0246) 23275353 PMC3631850

[34] Blum WF Alherbish A Alsagheir A Awwa AE Kaplan W Koledova E Savage MO. The growth hormoneinsulin-like growth factor-I axis in the diagnosis and treatment of growth disorders. Endocrine Connections 2018 7 R212–R222. (10.1530/EC-18-0099) 29724795 PMC5987361

[35] Green H Morikawa M Mxon T. A dual effector theory of growth-hormone action. Differentiation: Research in Biological Diversity 1985 29 195–198. (10.1111/j.1432-0436.1985.tb00316.x) 3908201

[36] Leung KC Doyle N Ballesteros M Waters MJ Ho KKY. Insulin regulation of human hepatic growth hormone receptors: divergent effects on biosynthesis and surface translocation. Journal of Clinical Endocrinology and Metabolism 2000 85 4712–4720. (10.1210/jcem.85.12.7017) 11134133

[37] Gahete MD Córdoba-Chacón J Lin Q Brüning JC Kahn CR Castaño JP Christian H Luque RM Kineman RD. Insulin and IGF-I inhibit GH synthesis and release in vitro and in vivo by separate mechanisms. Endocrinology 2013 154 2410–2420. (10.1210/en.2013-1261) 23671263 PMC3689283

[38] Xu J Ji S Venable DY Franklin JL Messina JL. Prolonged insulin treatment inhibits GH signaling via STAT3 and STAT1. Journal of Endocrinology 2005 184 481–492. (10.1677/joe.1.05977) 15749807

[39] Kaminski BA Goldsweig BK Sidhaye A Blackman SM Schindler T Moran A. Cystic fibrosis related diabetes: nutrition and growth considerations. Journal of Cystic Fibrosis 2019 18 (Supplement 2) S32–S37. (10.1016/j.jcf.2019.08.011) 31679727

[40] Prentice BJ Ooi CY Strachan RE Hameed S Ebrahimkhani S Waters SA Verge CF Widger J. Early glucose abnormalities are associated with pulmonary inflammation in young children with cystic fibrosis. Journal of Cystic Fibrosis 2019 18 869–873. (10.1016/j.jcf.2019.03.010) 31036487

[41] Mitchell DM Growth in patients with type 1 diabetes. Current Opinion in Endocrinology, Diabetes, and Obesity 2017 24 67–72. (10.1097/MED.0000000000000310) 27898589 PMC6097538

[42] Alicandro G Battezzati PM Battezzati A Speziali C Claut L Motta V Loi S Colombo C. Insulin secretion, nutritional status and respiratory function in cystic fibrosis patients with normal glucose tolerance. Clinical Nutrition 2012 31 118–123. (10.1016/j.clnu.2011.09.007) 21974813

[43] DeBoer MD Scharf RJ Leite AM Férrer A Havt A Pinkerton R Lima AA Guerrant RL. Systemic inflammation, growth factors, and linear growth in the setting of infection and malnutrition. Nutrition 2017 33 248–253. (10.1016/j.nut.2016.06.013) 27712965 PMC5193489

[44] Shoelson SE Lee J Goldfine AB. Inflammation and insulin resistance. Journal of Clinical Investigation 2006 116 1793–1801. (10.1172/JCI29069) 16823477 PMC1483173

[45] Battezzati A Bedogni G Zazzeron L Mari A Alicandro G Russo M Colombo C. Defective beta cell function measured during OGTT is a long term diabetes predictor in cystic fibrosis. Pediatric Pulmonology 2014 570 49.

[46] Pu MZMH Christensen-Adad FC Gonçalves AC Minicucci WJ Ribeiro JD Ribeiro AF. Insulin therapy in patients with cystic fibrosis in the pre-diabetes stage: a systematic review. Revista Paulista de Pediatria 2016 34 367–373. (10.1016/j.rpped.2015.12.010) 26994743 PMC5178124

[47] Moran A Pekow P Grover P Zorn M Slovis B Pilewski J Tullis E Liou TG Allen H & Cystic Fibrosis Related Diabetes Therapy Study Group. Insulin therapy to improve BMI in cystic fibrosis–related diabetes without fasting hyperglycemia: results of the cystic fibrosis related diabetes therapy trial. Diabetes Care 2009 32 1783–1788. (10.2337/dc09-0585) 19592632 PMC2752940

[48] Minicucci L Haupt M Casciaro R De Alessandri A Bagnasco F Lucidi V Notarnicola S Lorini R Bertasi S Raia V***et al***. Slow-release insulin in cystic fibrosis patients with glucose intolerance: a randomized clinical trial. Pediatric Diabetes 2012 13 197–202. (10.1111/j.1399-5448.2011.00810.x) 22060105

